# Association of TCRαβ^+^ double-negative T cells with the response to glucocorticoids in pediatric patients with immune thrombocytopenia

**DOI:** 10.3389/fimmu.2025.1645932

**Published:** 2025-07-30

**Authors:** Hui Chen, Xingjuan Xie, Jingyao Ma, Lingling Fu, Runhui Wu, Zhenping Chen

**Affiliations:** ^1^ Department of Clinical Laboratory Center, National Key Clinical Discipline of Pediatric Hematology, National Key Discipline of Pediatrics (Capital Medical University); Key Laboratory of Major Diseases in Children, Ministry of Education; Beijing Children’s Hospital, Capital Medical University, National Center for Children’s Health, Beijing, China; ^2^ Hematology Center, National Key Discipline of Pediatrics (Capital Medical University); Key Laboratory of Major Diseases in Children, Ministry of Education; Beijing Children’s Hospital, Capital Medical University, National Center for Children’s Health, Beijing, China

**Keywords:** double-negative T cell, glucocorticoid, high-dose dexamethasone, immune thrombocytopenia, pediatric

## Abstract

**Objectives:**

Pediatric primary immune thrombocytopenia (ITP) is an acquired autoimmune disease that can be partially restored by glucocorticoids. TCRαβ^+^CD4^−^CD8^−^ double negative T cells (TCRαβ^+^DNT) has been linked to the pathophysiology of ITP; however, the role of TCRαβ^+^DNT in response to high-dose dexamethasone (HD-DXM) is unclear. In this study, we aimed to explore the alteration in TCRαβ^+^DNT in ITP and the effect of HD-DXM on this subset.

**Materials and methods:**

Pediatric patients (aged <18 years) newly diagnosed with ITP were recruited for this retrospective study. Th1, Th17, Treg, and TCRαβ^+^DNT levels were measured by flow cytometry using specific antibodies. All patients received HD-DXM treatment and underwent periodic outpatient follow-up for 2-6 months. Patients were divided into the overall response (OR) and no response (NR) groups according to their responses to HD-DXM treatment.

**Results:**

We enrolled 130 pediatric patients with ITP (OR, 95 cases; NR, 35 cases) and 50 age- and sex-matched healthy controls. Compared with Th17-to Treg, Th17, and Th1, univariate analysis identified that the proportion of TCRαβ^+^DNT at baseline was more effective in predicting the response to HD-DXM (*P*<0.05). A significantly increased frequency of TCRαβ^+^DNT was found in patients with ITP compared to healthy controls (percentage of T cells: 1.31% vs. 1.00%, *P*<0.0001; percentage of lymphocytes: 0.76% vs. 0.68%, *P*=0.010). Patients in the NR group had a higher percentage of TCRαβ^+^DNT than the OR at the initial diagnosis (TCRαβ^+^DNT/T: 1.52% vs. 1.30%, *P*<0.01; TCRαβ^+^DNT/Lym: 0.84% vs. 0.72%, *P*<0.01). After treatment with HD-DXM, the elevated TCRαβ^+^DNT was effectively reduced in the OR group, but not in the NR group (TCRαβ^+^DNT/T: *P*<0.05; TCRαβ^+^DNT/Lym: *P*=0.001; TCRαβ^+^DNT counts: *P*<0.01).

**Conclusions:**

TCRαβ^+^DNT appears to play a significant role in the pathogenesis of pediatric ITP and may be involved in the immune response to HD-DXM. The correction of elevated TCRαβ^+^DNT in patients who respond to HD-DXM may provide a novel insight for immune therapy in pediatric ITP.

## 1 Introduction

Primary immune thrombocytopenia (ITP) is an acquired autoimmune hemorrhagic disease characterized by decreased platelet count due to enhanced platelet destruction and/or impaired platelet production. The estimated incidence of pediatric ITP is 2 to 5 per 100,000 children (1). Platelet production is a complex biological process that involves the generation and maturation of megakaryocytes, as well as the release of platelets. Platelets originate from megakaryocytes that express many immune receptors and cytokines in common with their “mother cells” (2). The classic pathogenesis of ITP is that antiplatelet antibodies mediate macrophage phagocytosis and the destruction of platelets. In recent years, the underlying mechanism of ITP has expanded to a more heterogeneous and complex immune pathophysiology including deficiency in T cell regulatory activity, altered megakaryocytic function (3–6). CD3^+^CD4^+^IFN-γ^+^ helper T lymphocyte (Th1) polarization is demonstrated by the increase in serum IFN-γ and IL-2 in ITP patients, and the Th1/Th2 ratio of T cells in the circulation and spleen is increased (7, 8). In addition, the percentage of CD3^+^CD4^+^IL-17^+^ helper T lymphocytes (Th17) to CD3^+^CD4^+^CD25^+^FOXP3^+^ regulatory T cells (Treg) is elevated at the newly diagnosed ITP stage (9). In our previous study, we used a range of machine learning algorithms in predicting the chronicity of pediatric ITP, and identified Th17, Th17-toTreg, Th1, and TCRαβ^+^CD4^−^CD8^−^ double negative T cells (DNT)/CD3^+^ T cells as the four most significant immune function indicators (10).

TCRαβ^+^DNT, a relatively small subpopulation comprising approximately 1-5% of all CD3^+^ T lymphocytes in peripheral blood. The most well-defined pathological condition characterized by the expansion of TCRαβ^+^DNT is autoimmune lymphoproliferative syndrome (ALPS), which is defined as exceeding 1.5% of total lymphocytes and/or >2.5% of CD3^+^ lymphocytes, according to current clinical guidelines (11, 12). In addition to serving as an important diagnostic marker for ALPS, TCRαβ^+^DNT has also been proven to play diverse functional roles in various diseases. First, TCRαβ^+^DNT can prevent the onset of and provide long-lasting protection against type 1 diabetes and graft versus host disease (GVHD) (13, 14). Achita et al. have confirmed that the infusion of allogeneic or xenogeneic TCRαβ^+^DNT alone does not cause GVHD due to the MHC-unrestricted characteristics (14). Second, multiple studies point to a critical anti-tumor activity exhibited by TCRαβ^+^DNT, producing IFN-γ, perforin and granzyme B, mediating the killing effect in hematologic malignancies and solid tumors (15–17). Lee and collaborators identified that TCRαβ^+^DNT demonstrated promising efficacy as an ex vivo therapeutic approach for the treatment of leukemia (17). Third, TCRαβ^+^DNT is capable of secreting proinflammatory cytokines, including IL-4, IL-17, and tumor necrosis factor-α (TNF-α), and plays a similar role to CD4^+^ Th cells. It contributes to several autoimmune diseases, such as systemic lupus erythematosus (SLE), psoriasis and Sjögren’s syndrome. TCRαβ^+^DNT can infiltrate the primary target organs, suggesting a direct and pathogenic role in tissue damage (18–20). Although the presence of DNT at sites of injury in autoimmunity strongly suggest their critical role in immune regulation, their potential immunomodulatory function in ITP remains poorly understood.

Glucocorticoids are the first-line treatment option for patients with ITP, especially dexamethasone, which is less toxic and more active against plasma cells. It has long been used in ITP for its global influence on the immune system by functionally suppressing both T- and B-cell reactions and restoring Tregs. High-dose dexamethasone (HD-DXM) treatment has been reported to restore the normal Th1/Th2 ratio, reduce Th17 cells, and increase Tregs (21–23). Moreover, evidence suggests that the intrinsic sensitivity of lymphocytes may be a major factor in determining their response to glucocorticoids (24, 25). However, some patients have a slow or no response to HD-DXM treatment, which implies the presence of other types of immune cells.

Here, we aimed to identify different immune function indicators that may contribute to the development and prognosis of newly diagnosed pediatric primary ITP, such as Th17, Th17-toTreg, Th1, and TCRαβ^+^DNT. In addition, we tried to further investigate the expression of blood TCRαβ^+^DNT in the pathogenesis of pediatric ITP, and analyze the association of the response to glucocorticoids with larger samples.

## 2 Materials and methods

### 2.1 Subjects

This retrospective study was approved by the Research Ethics Committee of Beijing Children’s Hospital, Capital Medical University Ethics Committee (approval No. 2018-k-97). Pediatric patients diagnosed with primary ITP between June 2018 and October 2022 were enrolled in this study at the Department of Hematology Center, Beijing Children’s Hospital. And age- and sex-matched healthy controls were recruited. Written informed consent was obtained from all volunteers and their parents.

The inclusion criteria were as follows: (1) patients newly diagnosed with ITP who met the criteria for primary ITP according to international guidelines (26); (2) patients aged < 18 years and ≥ 1 year; (3) patients with blood platelet counts less than 30× 10^9^/L; and (4) patients who planned to receive HD-DXM treatment, and were free from any previous ITP-specific therapy. Patients with secondary ITP were excluded from this study.

### 2.2 HD-DXM treatment methods and efficacy judgment

HD-DXM therapy entailed dexamethasone administration at a dose of 0.6 mg/kg/day, a maximum of 40 mg/day for 4 consecutive days, patients could receive additional cycle of HD-DXM if they did not respond. All patients received HD-DXM treatment, and their platelet counts were regularly monitored every week at local hospitals. Follow-up outpatient evaluations were conducted every four weeks at our institution, where the early therapeutic response was assessed at the end of the second month. A patient’s response to HD-DXM was categorized as either an overall response (OR) or no response (NR) after six months of treatment. OR was defined as any platelet count of at least 30 × 10^9^/L and at least doubling of the baseline count without bleeding, and NR was defined as a platelet count < 30 × 10^9^/L or < two-fold increase in the baseline platelet count or bleeding (26).

### 2.3 Immunostaining and flow cytometric analysis

Fresh EDTA-anticoagulated peripheral blood was collected from the enrolled patients at the time of initial diagnosis and 3–6 months after HD-DXM treatment. Routine testing of Th1, Treg, and Th17 cells was performed according to the standard operating procedure (SOP) of our lab (27).

For analysis of the TCRαβ^+^DNT, the following fluorescent-specific antibodies were used to analyze TCRαβ^+^DNT: anti-CD45-PerCP, anti-CD3-FITC, anti-CD4-PE-Vio770, anti-CD8-APC-Vio770, and anti-TCRαβ-APC (Miltenyi Biotec, Germany). The population of DNT was gated by CD4^−^CD8^−^ in CD3^+^TCRαβ^+^ cells, and the percentage of TCRαβ^+^DNT to lymphocytes or T cells was analyzed using the BD FACS Diva software. The number of TCRαβ^+^DNT per µL whole blood was calculated using the following formula: the number of TCRαβ^+^DNT subsets = the absolute number of lymphocytes × the percentage of TCRαβ^+^DNT subsets in lymphocytes. The absolute number of lymphocytes was obtained from the clinical routine blood tests of same blood collection.

### 2.4 Statistical analysis

GraphPad Prism 10.9 software was used to analyze all data. The normal distribution of the variables was examined by the Kolmogorov-Smirnov test. The difference between two groups was determined by t-test or non-parametric Mann-Whitney U tests. A *P*-value ≤ 0.05 was considered statistically significant.

## 3 Results

### 3.1 The clinical features of enrolled subjects

This study enrolled 130 ITP patients and 50 healthy controls. The demographic and clinical characteristics of ITP patients and controls are shown in **Table 1**. There were no significant differences in sex or age between patients and controls (*P*>0.05). Among all patients, 95 (73.08%) achieved OR, and 35 (26.92%) achieved NR after a 6-month follow-up. No significant differences in the baseline clinical features were found between the NR and OR groups.

**Table 1:** The clinical features of enrolled subjects.

Characteristics	ITP patients (n=130)		Healthy controls	*P* value
	OR	NR		
Cases	95	35	50	
Sex (M: F)	35: 60	18:17	27:23	>0.05
Median age; years (Q25, Q75)	4 (2, 7)	6 (3, 10)	5 (3.75, 6)	>0.05
Median Platelet counts; x 10^9^/L, (Q25, Q75)	8 (4, 15)	6 (3, 16)	316 (261, 349)	<0.0001

### 3.2 The proportion of TCRαβ^+^DNT at diagnosis in relation to the response to HD-DXM

We conducted an investigation using univariate analysis to assess the role of different immune indicators in the development and prognosis of pediatric ITP by examining the proportions of Th17-to Treg, Th17, Th1, and TCR-αβ^+^DNT at initial diagnosis. At the time of diagnosis in pediatric ITP, the clinical significance of the frequencies of Th17-to Treg, Th17, Th1, or TCR-αβ^+^DNT was demonstrated when compared to healthy controls (median 0.07 vs 0.22, *P*<0.0001; 0.48% vs. 0.95%, *P*<0.0001; 10.86% vs. 15.40%, *P*<0.0001; 1.31% vs. 1.00%, *P*<0.0001, respectively) (**Table 2**). However, to discriminate OR patients from NR ones receiving HD-DXM treatment, the percentage of TCRαβ^+^DNT exhibited a significantly greater difference (*P*<0.05) than Th17-to Treg (*P*>0.05), Th17 (*P*>0.05), and Th1 (*P*>0.05) (**Supplementary Figure 1**).

**Table 2:** The performance of immune function indicators in ITP patients and healthy controls (Median).

Indicators	ITP patients	Healthy controls	*P* value
Th17-to Treg	0.074	0.224	<0.0001
Th17%	0.48	0.95	<0.0001
Th1%	10.86%	15.40%	<0.0001
TCRαβ^+^DNT%	1.31%	1.00%	<0.0001

### 3.3 The percentage of TCRαβ^+^DNT was elevated in patients with ITP

**Figure 1:**
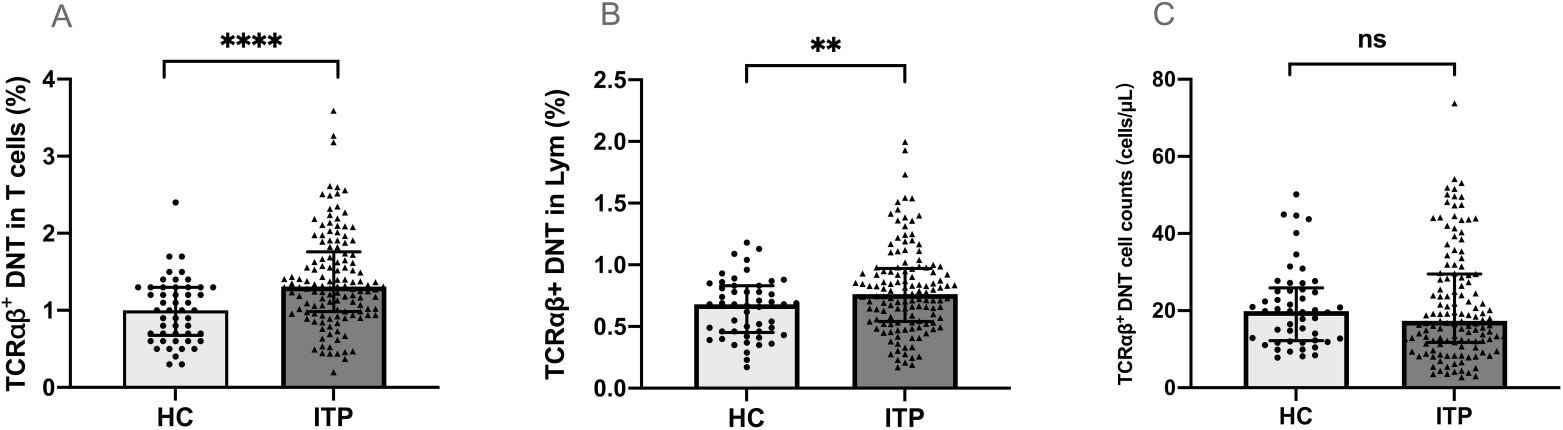
TCRαβ^+^DNT in pediatric patients with ITP and healthy controls. The percentage **(A, B)** and absolute number **(C)** of TCRαβ^+^DNT in ITP and healthy controls. Data were presented as Median (Q25, Q75) and analyzed by the Mann-Whitney U test. (**P<0.01; ****P<0.0001). DNT, double negative T cells; HC, healthy controls; ITP, immune thrombocytopenia; Lym, lymphocytes.

The percentage of TCRαβ^+^DNT in total CD3^+^T cells from peripheral blood was significantly higher in children with ITP than in healthy controls (median 1.31% vs. 1.00%, *P*<0.0001) (**Figure 1A**). The percentage of TCRαβ^+^DNT in lymphocytes was also higher in patients than in healthy controls (median 0.76% vs. 0.68%, *P*=0.010) (**Figure 1B**). However, there was no significant difference in TCRαβ^+^DNT counts between patients and controls (median 17.35 vs 19.80 cells/μL, *P*>0.05) (**Figure 1C**).

### 3.4 Higher frequency of TCRαβ^+^DNT were associated with poor response to HD-DXM in ITP patients

**Figure 2:**
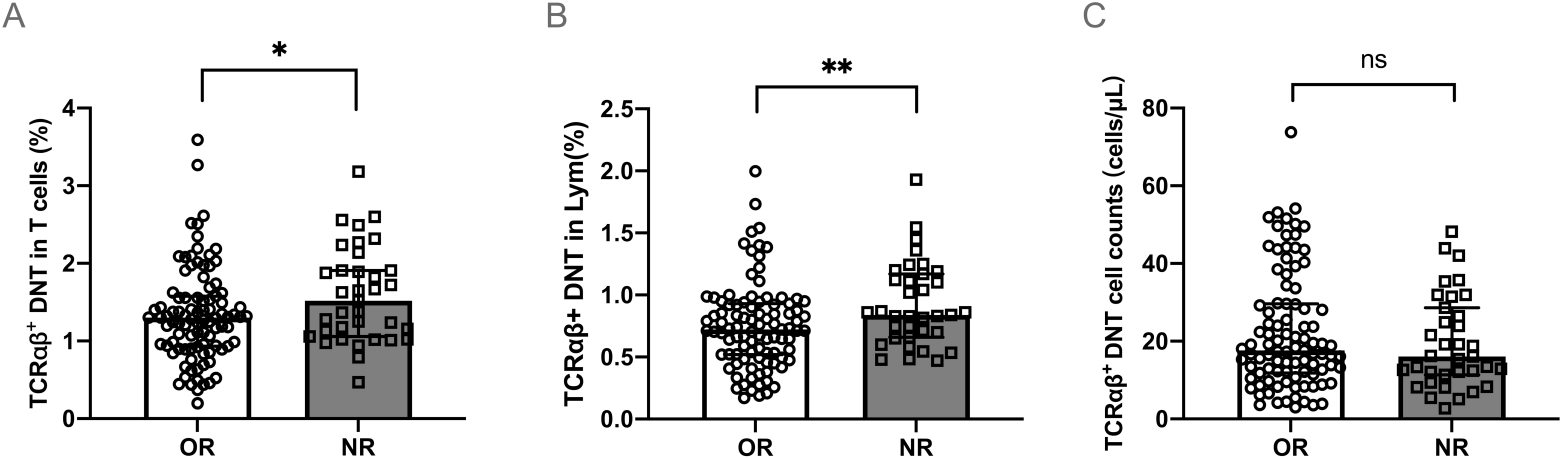
TCRαβ^+^DNT at initial diagnosis in different responses to HD-DXM. The percentage **(A, B)** or absolute number **(C)** of TCRαβ^+^DNT at initial diagnosis in OR and NR group. Data were presented as Median (Q25, Q75) and analyzed by the Mann-Whitney U test. (*P<0.05; **P<0.01). DNT, double negative T cells; OR, overall response; NR, no response; Lym, lymphocytes.

Compared to the OR group, patients in the NR group had a higher percentage of TCRαβ^+^DNT both in total T cells (median 1.52% vs. 1.30%, *P*<0.01) (**Figure 2A**) and lymphocytes (median 0.84% vs. 0.72%, *P*<0.01) (**Figure 2B**) at initial diagnosis. No notable difference was observed in the specific counts of TCRαβ^+^DNT cells between the OR and NR groups (*P*>0.05) (**Figure 2C**).

### 3.5 TCRαβ^+^DNT were reduced after HD-DXM treatment in OR group rather than NR group

**Figure 3:**
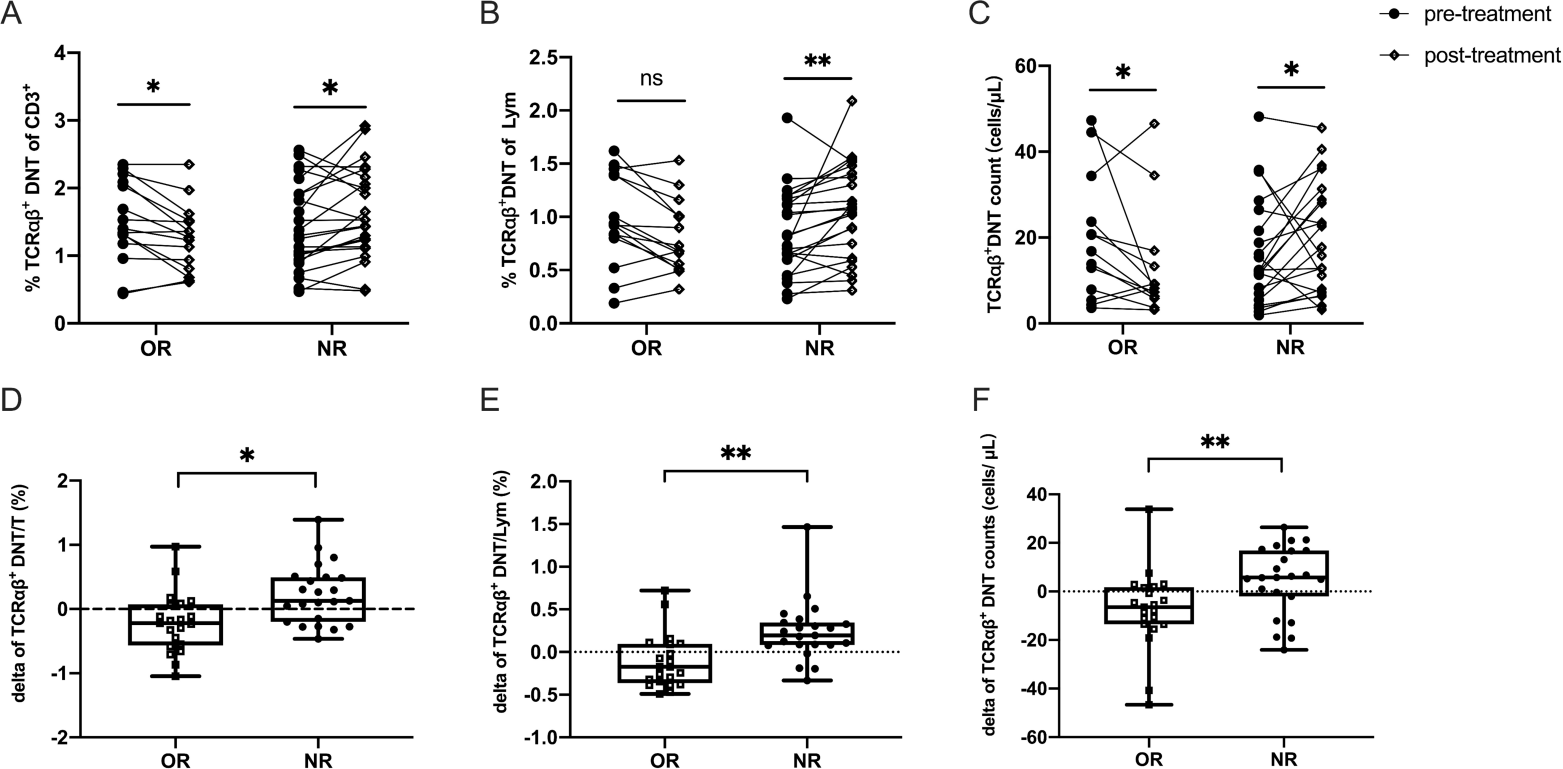
Efficacy of HD-DXM therapy in regulating the level of TCRαβ^+^DNT in pediatric patients with ITP. The levels of TCRαβ^+^DNT before and after HD-DXM treatment **(A-C)** in OR and NR group. The delta of proportion **(D, E)** and absolute number **(F)** of TCRαβ^+^DNT in OR and NR group (the delta value is equal to TCRαβ^+^DNT _post-t_ **-** TCRαβ^+^DNT _pre-t_). Data were presented as Mean ± standard deviation (SD) and analyzed by t-test. (*P<0.05; **P<0.01).

Next, to evaluate the effect of HD-DXM therapy on TCRαβ^+^DNT in pediatric ITP, we investigated the difference in the levels of TCRαβ^+^DNT between pre- and post-treatment. Peripheral blood samples were collected from 23 NR patients and 21 age- and sex-paired OR patients after 3-6 pulses of HD-DXM. The results showed that the level of TCRαβ^+^DNT presented different changing tendencies after HD-DXM intervention between the OR and NR groups (*P*<0.05) (**Figures 3A–C**). In the OR group, the mean difference in TCRαβ^+^DNT of total T cells between pre- and post-treatment was -0.214, which was lower than that in the NR group (mean = 0.224, *P*<0.01) (**Figure 3D**). The mean difference in TCRαβ^+^DNT of lymphocytes between pre- and post-treatment in the OR group was -0.114, and the mean difference in the NR group was 0.237 (*P*=0.001) (**Figure 3E**). Furthermore, the TCRαβ^+^DNT counts decreased in the OR group but increased in the NR group (-6.411 vs 5.781 cells/μL, *P*<0.01) (**Figure 3F**).

## 4 Discussion

Imbalanced T-lymphocyte subpopulations have been shown to be involved in the autoimmune pathogenesis of pediatric ITP (3). In our study, to explore the contribution of various immune indicators to the pathophysiology of ITP, the percentages of Th17-to Treg, Th17, Th1, and TCRαβ^+^DNT were analyzed in the peripheral blood of patients with ITP and healthy controls. Similar to what *Jingyao Ma* et al. found (10), we observed Th17-to Treg, Th17, Th1, and TCRαβ^+^DNT might participate in the onset of newly diagnosed ITP. Furthermore, we compared these indicators at the initial diagnosis between OR and NR to HD-DXM. Univariate analysis revealed that only the TCRαβ^+^DNT proportion was a key factor for ITP patients with ITP. Taken together, these data suggest that TCRαβ^+^DNT could play a significant role in both the development of ITP and the treatment outcome of HD-DXM.

TCRαβ^+^DNT, as a minor portion of αβ T cells that lack CD4 and CD8 markers, has been considered to contribute to many autoimmune diseases, such as autoimmune lymphoproliferative syndrome (ALPS), systemic Lupus Erythematosus (SLE), and psoriasis, along with their ability to help B cells produce autoantibodies and various pro-inflammatory cytokines including IL-17, IFN-γ, and IL-4 (11, 18, 19). However, in some studies, TCRαβ^+^DNT has been found to potentially exert an immunosuppressive function in specific diseases, such as graft versus host disease (GVHD) and type 1 diabetes (T1D), by suppressing B-cell proliferation and producing IL-10 (28, 29). Therefore, TCRαβ^+^DNT may play a pro-inflammatory or immunosuppressive role under different conditions. Based on the above findings, we conducted a comprehensive analysis of the alterations in TCRαβ^+^DNT in pediatric ITP and evaluated the impact of HD-DXM on this specific subset.

In the present study, we found that the level of TCRαβ^+^DNT was increased in pediatric patients with newly diagnosed ITP. This is consistent with prior studies that found that an abnormal proportion of total lymphocytes or CD3^+^ T lymphocytes probably contributed to the development of other autoimmune disorders (30, 31). It suggested that an elevated TCRαβ^+^DNT proportion may be involved in the immune imbalance of ITP.

HD-DXM, which is generally accepted as the most common first-line treatment for pediatric ITP, is thought to have an extensive effect on the immune system. According to previous studies, lymphocyte subsets can influence or predict their response to corticosteroids in patients with ITP, including abnormal CD4:CD8 ratios, higher Th17 levels, and lower Treg levels (21–23, 32). *Lu* et al. described a subtype of adenosine receptor CD39^+^Tregs that was decreased in patients with ITP, but after HD-DXM therapy, the responding patients showed an increase in Tregs and their ability to immunosuppress was improved (33). Additionally, previous studies have indicated that changes in platelet mass play a significant role in modulating Tregs (34, 35). One potential explanation for the reversal of Treg defects by increased platelet count is their ability to secrete substantial quantities of TGF-β1, a critical molecular responsible for inducing Tregs (36). Generally speaking, aberrant T cell differentiation and proliferation can be rectified by dexamethasone. However, no study has previously described the significance of TCRαβ^+^DNT in dexamethasone therapy. Our data showed that a higher TCRαβ^+^DNT level at initial diagnosis was associated with a poor response to HD-DXM. Patients with lower levels of TCR αβ^+^DNT had a sensitive response to glucocorticoids. This implies that TCRαβ^+^DNT might play a predictive role in HD-DXM efficacy.

Furthermore, the recovery of TCRαβ^+^DNT levels was more obvious after HD-DXM in the OR group than in the NR group. Yan’s et al. found that conventional methylprednisolone effectively reduced DNT cells, but not IL-17-producing DNT cells, in patients with autoantibody-associated vasculitis (AAV) (37). A study of primary Sjogren’s syndrome showed that expanded-disease-associated IL-17^+^ DNT cells may be resistant to dexamethasone treatment (38). These results suggest that there is a special subpopulation of DNT cells resistant to glucocorticoids, which may account for the unchanged level of TCRαβ^+^DNT after HD-DXM therapy in the NR group. Therefore, it might be helpful to predict the outcomes of HD-DXM by detecting the level of TCRαβ^+^DNT initially and after treatment.

Although this study utilized a larger sample size to explore the role of TCRαβ^+^DNT in pediatric ITP, some limitations still exist. First, the observation period was short, and the predictive role of TCRαβ^+^DNT in the long-term efficacy or prognosis of HD-DXM was not investigated. Second, this study design did not implement the recommended control-to-case ratio of 1:1. Despite these limitations, TCRαβ^+^DNT is involved in the development of pediatric ITP and affects the response to glucocorticoids.

## 5 Conclusions

In conclusion, our findings suggest that TCRαβ^+^DNT in the peripheral blood might contribute to T cell-mediated immune dysregulation in pediatric ITP and may be involved in immune responses to HD-DXM. We systematically analyzed the alterations in TCRαβ^+^DNT in pediatric patients with ITP. This demonstrates that the proportion of TCRαβ^+^DNT is higher in ITP patients than in healthy controls. The percentage of TCRαβ^+^DNT was significantly higher in NR patients at initial diagnosis. Interestingly, elevated TCRαβ^+^DNT can be effectively corrected by glucocorticoids in the OR group, rather than in the NR group. Overall, TCRαβ^+^DNT may play a significant role in the pathogenesis of pediatric ITP and influence the efficacy of HD-DXM therapy. Moreover, further studies are needed to explore the underlying mechanism of TCRαβ^+^DNT in pediatric patients with ITP.

## Data Availability

The raw data supporting the conclusions of this article will be made available by the authors, without undue reservation.

## Supplementary material

Supplementary Figure 1 The performance of various immune function indicators at initial diagnosis with differential efficacy in pediatric ITP receiving HD-DXM treatment. (**P*<0.05).
