# Supplementary figures and images for: Association of TCRαβ+ double-negative T cells with the response to glucocorticoids in pediatric patients with immune thrombocytopenia
Source: Front Immunol. 2025 Jul 30;16:1645932. doi: 10.3389/fimmu.2025.1645932 (PMC12343612; doi:10.3389/fimmu.2025.1645932)

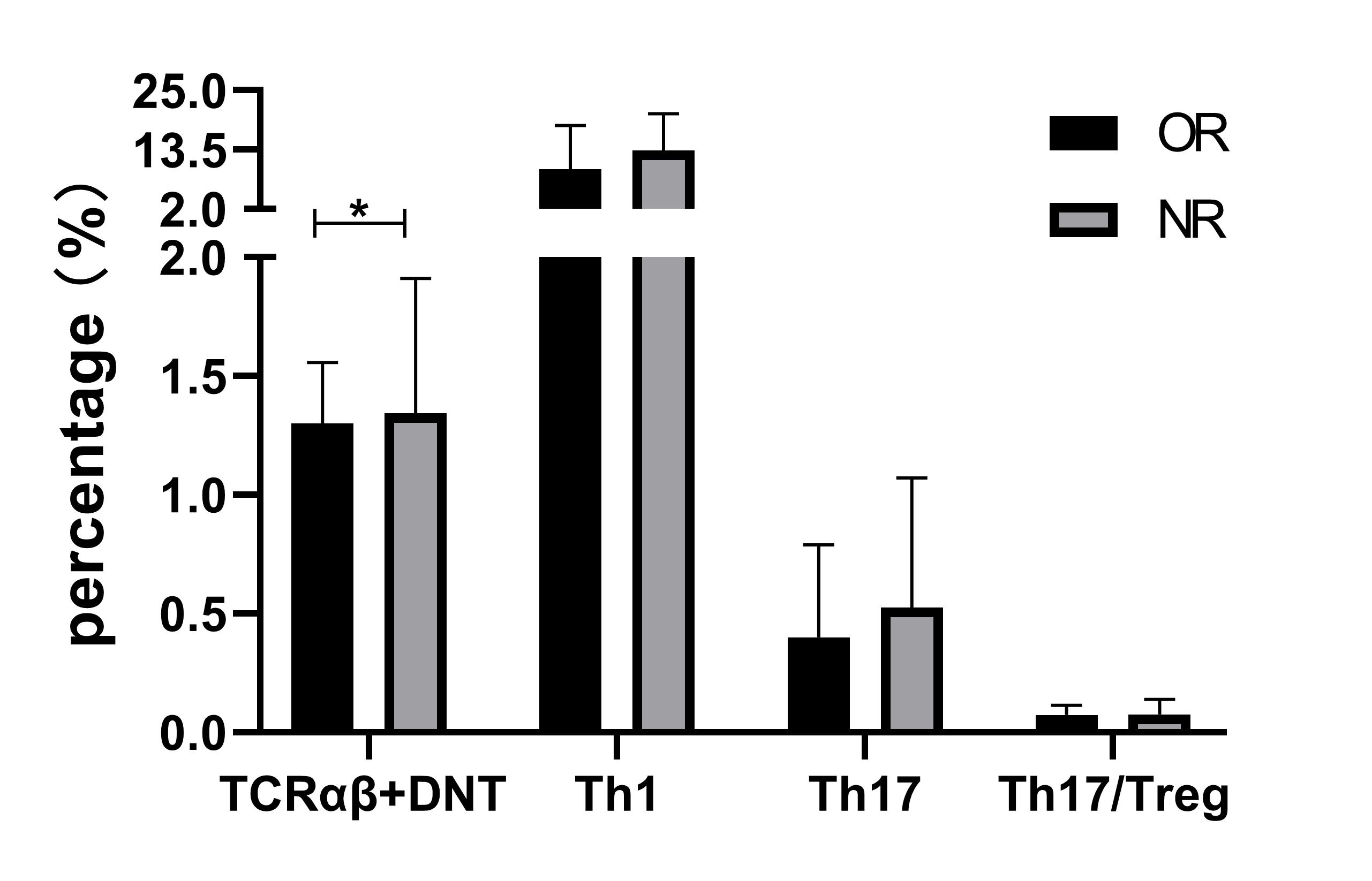

Supplement: Supplementary Figure 1 — The performance of various immune function indicators at initial diagnosis with differential efficacy in pediatric ITP receiving HD-DXM treatment. (*P<0.05). [file Image1.jpeg]
